# Clinical outcome of neoadjuvant chemoradiation in rectal cancer treatment

**DOI:** 10.1097/MD.0000000000027366

**Published:** 2021-09-24

**Authors:** Weerapat Suwanthanma, Saowanee Kitudomrat, Chakrapan Euanorasetr

**Affiliations:** Division of General Surgery, Department of Surgery, Faculty of Medicine, Ramathibodi hospital, Mahidol University, Bangkok, Thailand.

**Keywords:** neoadjuvant chemoradiation, pathological complete response, rectal cancer

## Abstract

To determine the clinical and pathological outcome of locally advanced rectal cancer patients treated with neoadjuvant chemoradiation (chemoradiotherapy [CRT]) followed by curative surgery and to identify predictive factors of pathological complete response (pCR).

Locally advanced rectal cancer patients undergoing CRT followed by curative surgery from January 2012 to December 2017 were included. Patient's demographic data, pretreatment tumor characteristics, type of CRT regimens, type of surgery, postoperative complications, pathological reports and follow up records were analyzed. Univariate and multivariate analyses were applied to identify predictive factors for pCR. Five-year disease free and overall survival were estimated by Kaplan–Meier method and compared between pCR and non-pCR groups.

A total of 85 patients were analyzed. Eighteen patients (21.1%) achieved pCR. The sphincter-saving surgery rate was 57.6%. After univariate analyses, tumor length >4 cm (*P* = .007) and positive lymph nodes (*P* = .040) were significantly associated with decreased rate of pCR. Complete clinical response was significantly associated with higher rate of pCR (*P* = .015). Multivariate analyses demonstrated that tumor length >4 cm (*P* = .010) was significantly associated with decreased rate of pCR. After a median follow-up of 65 months (IQR 34–79), the calculated 5-year overall survival and disease-free survival rates were 81.4% and 69.7%, respectively. Patients who achieved pCR tend to had longer 5-year disease-free survival (*P* = .355) and overall survival (*P* = .361) than those who did not.

Tumor length >4 cm was associated with decreased rate of pCR in locally advanced rectal cancer who had CRT followed by surgery. Longer waiting time or more intense adjuvant treatment may be considered to improved pCR and oncological outcomes.

## 1 Introduction

Multimodalities treatment with preoperative concurrent chemoradiotherapy (CRT) followed by total mesorectal excision (TME) were used as standard treatment for locally advanced mid and low rectal cancer to improved oncological outcomes in term of local recurrence and overall survival.^[1–6]^ Further regression of tumor after CRT increases the possibility for local control and sphincter preservation.^[7,8]^ Previous studies showed that 15% to 27% of patients following CRT and rectal surgery would get the maximum benefit and had a pathological complete response (pCR).^[6,9,10]^ The patients who achieved pCR experienced more favorable oncological outcome compared with patients who not achieved.^[11,12]^ However, there were some patients who did not response well after CRT and did not get benefit from these treatments. Therefore, it would be better if there were some methods to predict the response CRT before starting the treatment.

Few studies have reported some conflicting result of clinical predictive factors for pCR, including tumor size,^[13]^ tumor size from calculation by volumetry method,^[14]^ nodal stage,^[15]^ and pretreatment carcinoembryonic antigen (CEA) level.^[16]^ Tumor length, as measured by computerized tomography scan or magnetic resonance imaging, is one of the routine clinical parameters collected in management of rectal cancer. Data on tumor length as a predictive factor of pCR were previously mentioned in esophageal cancer after treatment with neoadjuvant chemoradiotherapy^[17]^ However, previous studies on tumor length as a predictive factor for pCR in rectal cancer are scarce and inconsistent.^[18–20]^ The lack of consistency may be attributable to the limited number of studies and small number of patients who achieved pCR. Therefore, there was no consensus on whether tumor length should be use as one of the clinically feasible predictive factors for pCR.

In our study, we conducted a retrospective cohort to determine clinical outcome and identify rate and clinical predictive factors associated with pCR after CRT in the patients with locally advanced rectal cancer at our tertiary referral center. We further examine if the tumor length and its appropriate cut-point, measured preoperatively, can adequately predict pCR.

## 2 Materials and methods

### 2.1 Patients

**Figure 1:**
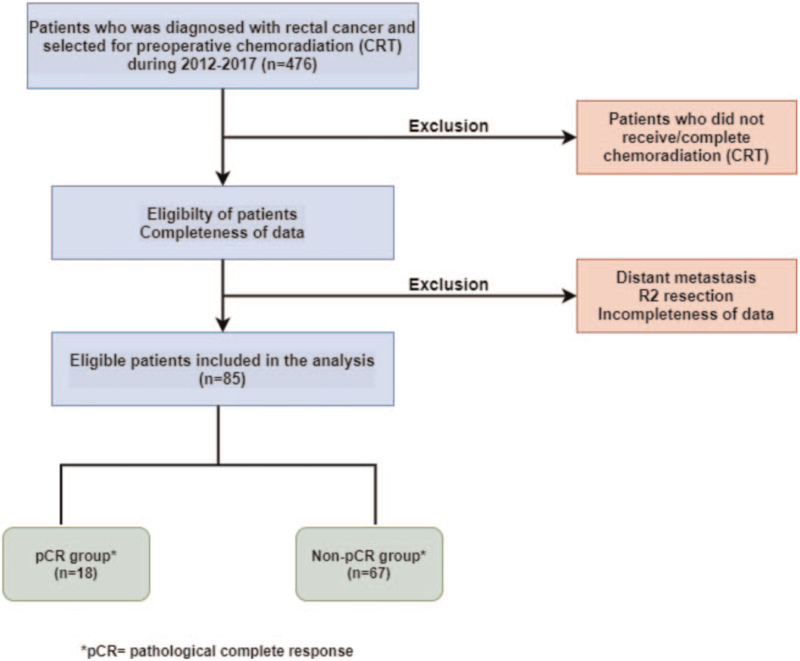
Study flow diagram. pCR = pathological complete respone, R2 = gross residual disease.

All 476 rectal cancer patients were identified in the tumor registry of Ramathbodi Hospital from January 2012 to December 2017. Eighty-five primary rectal adenocarcinoma patients treated with CRT followed by curative intent rectal resection with total mesorectal excision who met the following criteria were included in our study: histopathologically confirmed adenocarcinoma, age ≥18 years old, distal extent of tumor <15 cm above the anal verge, clinical stage of T3/4 or positive lymph nodes. Patients who refused surgery, who had evidence of distant metastasis, who did not receive CRT, who had R2 resection and who had incompleteness of data were excluded (Fig. 1).

We collected all available baseline clinical characteristics before CRT: age, gender, preoperative biopsy results. Initial tumor stage was assessed before CRT by Computerized Tomography (CT) of Chest and whole abdomen, Magnetic Resonance Imaging (MRI) of pelvis. All patients were evaluated with a physical examination, colonoscopy or flexible sigmoidoscopy. Tumor height and length were estimated by rectal examination and endoscopy, and the tumor thickness and tumor length were measured with CT and MRI. CEA levels were determined before, after the completion of CRT and after the operation.

The study was reviewed and approved by ethical committee on human rights related to research involving subjects (ID 08-58-46). The requirement for informed consent was waived because of retrospective study design.

### 2.2 Treatment

All patients received Three-dimension conformal radiotherapy (3D-CRT). The delivered target dose of protocol was 45 Gy to the rectal tumor with a boost of 5.4 Gy limited to the mesorectum. A median radiation dose of 50.4 Gy (range, 46–54 Gy) over a mean duration of 5.6 weeks was given. 3 patients (5.5%) also received an additional boost restricted to the tumor, receiving up to a total of 54 Gy. In majority of patients, concurrent 5-fluorouracil (5-FU)-based therapy was administered either as a bolus infusion 5FU+leucovorin (LV) during the initial 5 weeks of radiotherapy or as a continuous intravenous infusion throughout the radiotherapy. In few patients, XELOX regimens or Xeloda were used. Evaluation of clinical response was performed using one of these or in combination of digital rectal examination, flexible sigmoidoscopy, CT scan or pelvic MRI which was determined by the multidisciplinary team. After proper waiting time from last dose of radiation, all patients in the study underwent curative intent resection either low anterior resection (LAR), ultraLAR or abdominoperineal resection (APR) with TME by our colorectal surgeons. All available data of clinical and surgical outcomes and complications were collected.

### 2.3 Pathologic staging

The grade of the tumor was assessed from the initial tumor biopsy and grouped into 1 of 3 categories: high, moderate, poor, and unidentified differentiation. The maximum tumor size measured by the maximum size of viable residual tumor cells in centimeters was documented. pCR was defined as the absence of viable tumor cells in the rectal wall and in any of the resected lymph nodes. The presence of acellular mucin at the previous tumor site or in lymph nodes was not considered as residual viable cancer cells. Patients with microscopic residual disease, defined as only a few clusters of viable malignant residual cells in the specimen (<1 mm), were in non-complete pathological response group. All pathological result of patients was collected and reported according to the eighth edition from the American Joint Committee on Cancer.

### 2.4 Statistical analyses

Statistical analysis was performed by using STATA (Version 14; Stata Corp LP). Analysis of patient characteristics data were compared between 2 groups (pCR and non-pCR) using the Student's *t* test or Mann–Whitney for continuous variables, and Chi-square or Fisher's exact test for categorical variables. Univariate and multivariate analyses were performed by using the logistic regression model, Odd ratios (OR) and 95% confidence intervals (CI) were calculated to identify predictors for pCR. Disease-free survival (DFS) and overall survival (OS) were estimated using the Kaplan–Meier method and differences between survival curves were determined by using the log-rank test. A *P-*value <.05 was considered statistically significant.

## 3 Results

### 3.1 Clinical, disease and CRT characteristics

A total of 85 patients were included. The mean age was 59 years (range, 24–84 years). Clinical data, disease variables and CRT characteristics are presented in Table 1. For comparing of pCR and non-pCR groups, no statistically difference was found in gender, location of tumor, type of preoperative biopsies, clinical N stage, chemotherapy regimens, waiting time to surgery and CEA level before and after CRT. Our patients were predominantly male (68.9%). pCR groups were significantly older (*P* = .049). The mean pretreatment tumor length among pCR groups was significantly shorter compared with non-pCR groups (3.8 cm vs 5.4 cm). When using 4 cm of pretreatment tumor length as a cut-point, there was significant difference of pCR rate between pretreatment tumor length ≤4 and >4 cm group (*P* = .006). Most patient (83.5%) had pretreatment clinical T3 stage. There were 53 patients (62.4%) who had clinical N staging positive from imaging.

**Table 1:** Descriptive characteristics.

Variables	Total (n = 85)	Non-pCR (n = 67)	pCR (n = 18)	*P*
Age (year), mean±SD	59.3 ± 10.9	58.1 ± 11.3	63.8 ± 8.4	.049
Gender, n (%)				
Male	56 (68.9)	44 (65.7)	12 (66.7)	.937
Female	29 (34.1)	23 (34.3)	6 (33.3)	
Location (cm from anal verge), mean ± SD	5.6 ± 2.7	5.5 ± 2.8	5.9 ± 2.4	.606
Preoperative biopsy, n (%)				
Well differentiation	16 (18.8)	15 (22.4)	1 (5.6)	.457
Moderately differentiation	54 (63.5)	41 (61.2)	13 (72.2)	
Poorly differentiation	8 (9.4)	6 (8.9)	2 (11.1)	
Unknown differentiation	5 (5.9)	3 (4.5)	2 (11.1)	
Fragment of dysplastic cell	1 (1.2)	1 (1.5)	0	
Tubular adenoma, high grade dysplasia	1 (1.2)	1 (1.5)	0	
Tumor length (cm), mean ± SD	5.1 ± 2.3	5.4 ± 2.4	3.8 ± 1.0	.000
≤ 3 cm	14 (16.5)	8 (11.9)	6 (33.3)	.066
> 3 cm	71 (83.5)	59 (88.1)	12 (66.7)	
≤ 4 cm	33 (38.8)	21 (31.3)	12 (66.7)	.006
> 4 cm	52 (61.2)	46 (68.7)	6 (33.3)	
cT stage, n (%)				
Stage 2	4 (4.7)	1 (1.5)	3 (16.7)	.028
Stage 3	71 (83.5)	57 (85.1)	14 (77.8)	
Stage 4	10 (11.8)	9 (13.4)	1 (5.6)	
cN stage, n (%)				
Negative	32 (37.6)	24 (35.8)	8 (44.4)	.503
Positive	53 (62.4)	43 (64.2)	10 (55.6)	
Chemo regimens, n (%)				
5FU + LV	69 (81.2)	52 (77.6)	17 (94.4)	.362
Xeloda	13 (15.3)	12 (17.9)	1 (5.6)	
XELOX	3 (3.5)	3 (4.5)	0	
Waiting time to surgery (day), mean+SD	67.8 ± 23.5	63.2 ± 20.6	74.1 ± 31.9	.333
≤8 weeks	22 (25.9)	18 (26.9)	4 (22.2)	.690
>8 weeks	63 (74.1)	49 (73.1)	14 (77.8)	
≤10 weeks	61 (71.8)	50 (74.6)	11 (61.1)	.258
>10 weeks	24 (28.2)	17 (25.4)	7 (38.9)	
≤11 weeks	70 (82.4)	57 (85.1)	13 (72.2)	.293
>11 weeks	15 (17.6)	10 (14.9)	5 (27.8)	
PreCCEA, median (IQR) n = 78	7.7 (3.1, 22.3)	8.5 (3.7, 22.3)	3.5 (2.6, 22.9)	.250
≤5 ng/dL	32 (41.0)	23 (37.1)	9 (56.3)	.165
>5 ng/dL	46 (59.0)	39 (62.9)	7 (43.7)	
PostCCEA, median (IQR) n = 63	3.7 (2.3, 5.4)	3.8 (2.3, 5.6)	3.6 (2.6, 5.3)	.865
≤3 ng/dL	25 (39.7)	20 (41.7)	5 (33.3)	.565
>3 ng/dL	38 (60.3)	28 (28.3)	10 (66.7)	
Postoperative CEA, median (IQR) n = 83	2.0 (1.2, 3.0)	2.0 (1.2, 2.8)	2.2 (1.3, 3.6)	.654

cN = clinical lymph node, IQR = interquartile range, pCR = pathological complete response cT = clinical tumor, PostCCEA = postchemoradiotherapy CEA, PreCCEA = prechemoradiotherapy CEA.

Majority of chemotherapy given in both groups was 5-FU+LV regimen (81.2%). The overall mean interval time between radiation and surgery was 67.8 days (range, 27–186 days). pCR group patients had longer waiting time to surgery but this difference did not reach statistically significance. However, in the small subgroup of patients with longer waiting time >11 weeks, 5 from 10 (50%) of them achieved pCR.

### 3.2 Clinical response, surgical outcomes and complications

Table 2 gives results of clinical response, surgical outcome and postoperative complications. Type of operations and complications were similar between both groups. Nearly half of patients (45.8%) were assessed for clinical response, mainly from the patients treated since the year 2015 to 2017. Response of CRT seen by endoscopy, partial or complete, had significant association with pCR (*P* = .001). The complete clinical response subgroup had achieved higher rate of pCR (5 from 6 patients, 83.3%) compared to partial clinical response subgroup (2 from 29 patients, 6.9%).

**Table 2:** Clinical response, surgical outcomes and pathological results.

Variables	Total (n = 85)	Non-pCR (n = 67)	pCR (n = 18)	*P*
Clinical Response, n (%)				
No	1 (1.2)	1 (1.5)	0	.001
Not assessed	47 (55.3)	36 (53.7)	11 (61.1)	
Partial clinical response	31 (36.5)	29 (43.3)	2 (11.1)	
Complete clinical response	6 (7.0)	1 (1.5)	5 (27.8)	
Operation, n (%)				
LAR	41 (48.2)	32 (47.8)	9 (50.0)	.194
ultraLAR	8 (9.4)	4 (5.9)	4 (22.2)	
APR	22 (25.9)	19 (28.4)	3 (16.7)	
LAR with end colostomy	14 (16.5)	12 (17.9)	2 (11.1)	
Complication, n (%)				
No	69 (81.2)	52 (77.6)	17 (94.4)	.173
Yes	16 (18.8)	15 (22.4)	1 (5.6)	
SSI	6 (7.1)	6 (9.0)	0	
SSI (perineal)	5 (5.9)	4 (5.9)	1 (5.6)	
Presacral collection	3 (3.4)	3 (4.5)	0	
Anastomotic stricture	1 (1.2)	1 (1.5)	0	
Urine retention	1 (1.2)	1 (1.5)	0	
Specimen group, n (%)				
Well differentiation	7 (8.2)	7 (10.5)	–	–
Moderately differentiation	48 (56.5)	48 (71.6)	–	
Poorly differentiation	3 (3.5)	3 (4.5)	–	
Residual tumor, cannot classify	8 (9.4)	8 (11.9)	–	
Mucinous type	1 (1.2)	1 (1.5)	–	
Distal margin, mean ± SD	2.9 ± 2.1	2.9 ± 2.2	2.9 ± 1.5	.991
T downstaging, n (%)				
No	25 (29.4)	25 (37.3)	0	.002
Yes	60 (70.6)	42 (62.7)	18 (100)	
ypT stage, n (%)				
Stage 0	19 (22.4)	1 (1.5)	18 (100)	.000
Stage 1	1 (1.2)	1 (1.5)	0	
Stage 2	22 (25.9)	22 (32.8)	0	
Stage 3	43 (50.6)	43 (64.2)	0	
N downstaging, n (%)				
No	55 (64.7)	45 (67.2)	10 (55.6)	.360
Yes	30 (35.3)	22 (32.8)	8 (44.4)	
Nodal status, n (%)				
Negative	57 (67.1)	41 (61.2)	16 (88.9)	.026
Positive	28 (32.9)	26 (38.8)	2 (11.1)	
1–3 lymph nodes	22 (78.6)	20 (76.9)	2 (100)	.999
>3 lymph nodes	6 (21.4)	6 (23.1)	0	
Number of lymph nodes harvested, mean±SD	14.1 ± 9.0	14.9 ± 9.2	11.1 ± 7.9	.496
Angiolymphatic invasion, n (%) n = 68				
Absence	49 (72.1)	39 (67.2)	10 (100)	.052
Presence	19 (27.9)	19 (32.8)	0	
Perineural invasion, n (%) n = 68				
Absence	55 (80.9)	45 (77.6)	10 (100)	.189
Presence	13 (19.1)	13 (22.4)	0	

APR = abdominoperineal resection, LAR = low anterior resection, N = node, pCR = pathological complete response, SSI = surgical site infection, T = tumor, ultraLAR = ultra low anterior resection, ypT = pathological T stage after neoadjuvant chemoradiation.

All patients underwent oncologic surgery with TME techniques. Of the 85 cases, 41 (48.2%) underwent LAR, 8 (9.4%) proceeded with ultraLAR and 36 (42.4%) had either APR or LAR with end colostomy. Of the 63 cases who had LAR, 50 (77.7%) of them had anastomosis while 14 (22.3%) of them the anastomoses were abandoned due to poor sphincter function or oncological reason. The overall sphincter-saving surgery rate was 57.6%.

There was no mortality within 30 days after surgery. The overall complications rate was 18.8%. Six (7.1%) patients developed superficial surgical site infection. Three (3.4%) patients had presacral collection which subsequently required percutaneous drainage for resolution. One (1.8%) patient had anastomosis stricture. In 22 patients who had APR, 5 (22.7%) of them had perineal wound infection and 1 patient had postoperative urinary retention.

### 3.3 Pathological outcome

Pathological data was available for 85 patients (Table 2). Of these, 18 (21.1%) patients achieved pCR. Histologic types of cancer, margin status, N (node) downstaging, number of lymph nodes harvested, lymphovascular invasion and perineural invasion were similar between both groups. Of all patients who had residual tumor, tumor histology mainly was moderately differentiated histology (71.6%).

Overall N downstaging rate was 35.3% which was lower compared to T (tumor) downstaging (70.6%). Total number of lymph nodes harvested was not difference in pCR (11.1 nodes) and non-pCR (14.9) groups (*P* = .496). Of the pathologic variables, T downstaging and positive lymph nodes status were correlated with pCR.

### 3.4 Oncological and survival outcome

**Figure 2:**
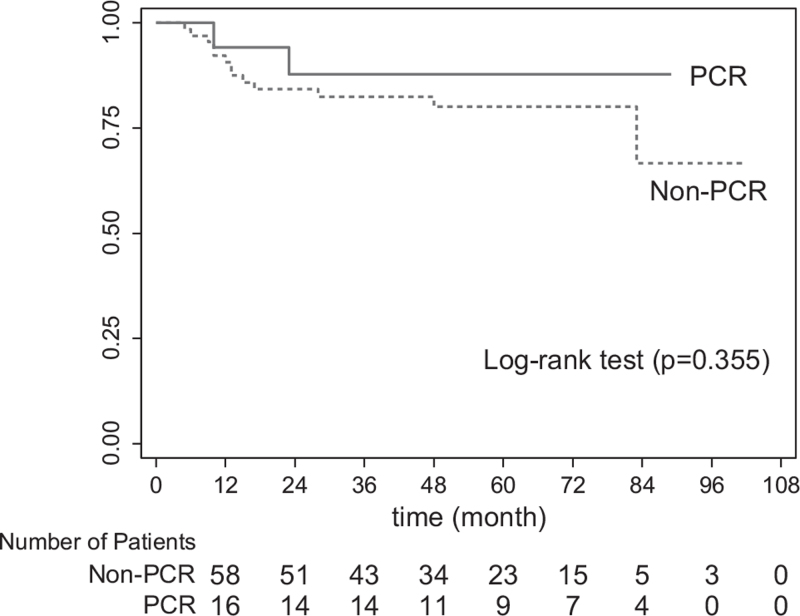
Five-year disease-free survival in patients with pCR vs those with non-pCR. pCR = pathological complete response.

**Figure 3:**
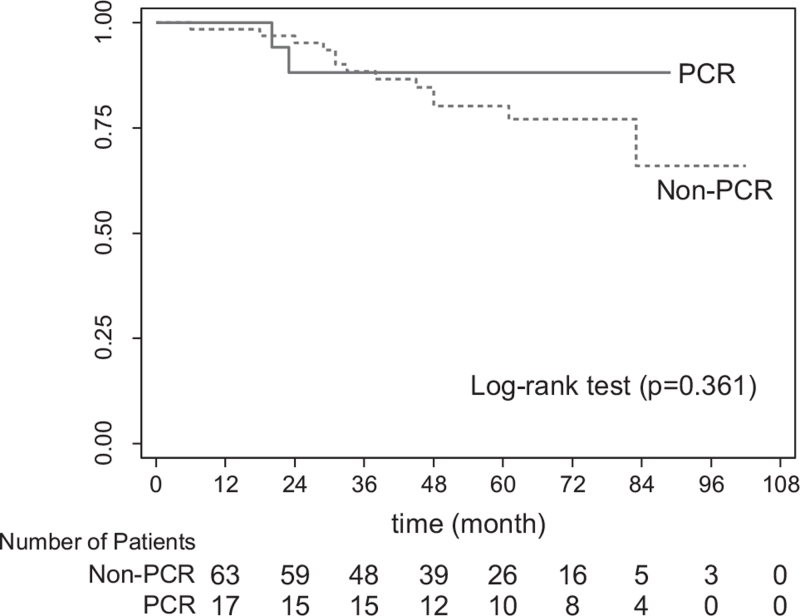
Overall 5-year survival in patients with pCR vs those with non-pCR. pCR = pathological complete response.

Table 3 summarizes the oncological outcome. The mean follow-up time was 65 months (IQR 34–79). At the time of survival analysis, 15 patients (17.6%) had died. Two patients died from non-rectal cancer related causes (1 from angiosarcoma of spleen and another from bleeding neurofibromatosis at back). Overall recurrence rate was 30.6%. Most common site of recurrence was lung (34.6%). More local recurrence was occurred in non-pCR group (9.1%), but this difference did not reach statistically significance. The calculated 5-year overall survival (OS) and DFS rates were 81.4% and 69.7%, respectively. The Kaplan–Meier method revealed that the number of patients who achieved pCR tend to had higher DFS and OS rates than those who did not, even without statistically significant (*P* = .355 and *P* = .361, respectively; Figs. 2 and 3).

**Table 3:** Oncological outcomes.

Variables	Total (n = 85)	Non-pCR (n = 67)	pCR (n = 18)	*P*
Follow up time (month), median (IQR)	65 (34, 79)	61 (31, 78)	74 (64, 88)	.107
Recurrence, n (%)				
No	59 (69.4)	45 (67.2)	14 (77.8)	.386
Yes	26 (30.6)	22 (32.8)	4 (22.2)	
Liver	5 (19.2)	5 (22.7)	0	.560
Lung	9 (34.6)	6 (27.3)	3 (75.0)	
Local recurrence	2 (7.7)	2 (9.1)	0	
> 1 locations	6 (23.1)	5 (22.7)	1 (25.0)	
Unknown	4 (15.4)	5 (18.2)	0	
Death, n (%)				
No	70 (82.4)	54 (80.6)	16 (88.9)	.509
Yes	15 (17.6)	13 (19.4)	2 (11.1)	
Death from disease	13 (15.3)	11 (16.4)	2 (11.1)	
Death from other causes	2 (2.4)	2 (3.0)	0	

IQR = interquartile range, pCR = pathological complete response.

### 3.5 Predictive factors for pCR

Four clinical predictors of pCR (age, tumor length, clinical response, and nodal status) were selected into the evaluation system. The univariate analysis indicated that Tumor length >4 cm (OR, 0.228; 95%CI, 0.08–0.69; *P* = .009) and positive lymph nodes status (OR, 0.197; 95%CI, 0.04–0.93; *P* = .04) were significantly and negatively associated with pCR. In contrast to tumor length and positive lymph node status, complete clinical response (OR, 16.364; 95% CI, 1.72–155.36; *P* = .015) was significantly correlated with increased pCR (Table 4).

**Table 4:** Univariate analyses on predicting factors for pCR.

	Univariate analyses	
Variables	OR (95%CI)	*P*
Age (year)	1.058 (0.99–1.12)	.054
Tumor length (cm)	0.582 (0.39–0.86)	.007
≤ 4 cm	1	
> 4 cm	0.228 (0.08–0.69)	.009
Clinical response		
Partial clinical response	0.226 (0.05–1.10)	.066
Complete clinical response	16.364 (1.72–155.36)	.015
Nodal status		
Negative	1	
Positive	0.197 (0.04–0.93)	.040

CI = confidence interval, OR = odds ratio, pCR = pathological complete response.

The multivariate analysis revealed that only tumor length >4 cm (OR, 0.134; 95%CI, 0.03–0.59; *P* = .008) was a significant predictor of decreased rate of pCR. Although it did not reach statistical significance, there was a trend for complete clinical response and node negative patients to achieve pCR (Table 5).

**Table 5:** Multivariate analyses on predicting factors for pCR.

	Multivariate analyses	
Variables	OR (95%CI)	*P*
Tumor length (cm)		
≤ 4 cm	1	
> 4 cm	0.158 (0.04–0.65)	.010
Clinical Response		
Complete clinical response	8.611 (0.67–110.6)	.098
Nodal status		
Negative	1	
Positive	0.307 (0.09–1.11)	.071

CI = confidence interval, OR = odds ratio, pCR = pathological complete response.

## 4 Discussion

This study described clinical, surgical and pathological factors associated with pathological response and oncological outcomes in 85 locally advanced rectal cancer treated with CRT followed by surgery over a 6-year period at the tertiary hospital in Thailand. The overall pCR rate of 21.1% is comparable with previous studies.^[6,9,10]^ Our result demonstrated that tumor length ≥4 cm was found to be predictive factor with decreased pCR on univariate and multivariate analyses.

Neoadjuvant chemoradiation (CRT) has been the standard of care for patients with locally advanced rectal cancer because it contributes high rate of local control and sphincter preservation^[7,21]^ which resulted from effect of tumor shrinkage.^[11,22]^ Patients who had CRT together with good rectal cancer surgery (TME) yield better oncological result by decrease local recurrence and increase DFS and OS.^[9,11,12]^ After complete CRT, patients were re-evaluated for possible clinical complete response (cCR), which defined as no gross tumor was seen in physical examination, endoscopy and post-CRT imaging. From flexible sigmoidoscopy, cCR was defined as area of scarring without gross tumor at the rectal mucosa. Patients who achieve cCR can selectively be candidates in watch and wait protocol. Even low endoscopic evaluation rate after CRT (38 patients, 44.7%) in our study, 6 patients (15.7%) achieve cCR and 31 patients (81.5%) achieve incomplete clinical response.

Waiting for the highest degree of tumor downstaging after CRT is of clinical relevance, as this will optimize the chance of an R0 resection and sphincter-saving surgery. Furthermore, after waiting for cCR, some subgroups of patients can also achieve pCR. pCR, defined by no residual tumor cell found in pathological specimens, is a crucial predictive factor associated with favorable oncological outcome, which was previously demonstrated in many studies.^[9,23,24]^ The pCR rate in this study was 21.1%, which was comparable to other studies ranged between 10% and 26%.^[9,25]^

There are numerous techniques to increase pCR rate. One of the most important predictors previously studied for increase pCR rate is waiting time interval after CRT to surgery. However, the strategy of increased waiting time to increase pCR requires a balance between allowing sufficient time for the maximal effects of CRT to be achieved and not allowing too much time so the tumor can repopulate. Lyon R90–01 trial, published in 1999 was the only randomized controlled trial to examine the time interval to surgery. In this study, a total of 210 patients with rectal cancer were randomized between surgery after a short (<2 weeks) and long (6–8 weeks) interval from the last day of CRT. Their result showed that the longer interval was associated with a significant higher patients group with ypT0–1 in resected specimens, but not pCR.^[26]^ Moore et al reported in 2004 that trend toward increased pCR rates and downstaging with increased waiting time interval. However, sphincter preservation is not increased.^[27]^ Large retrospective cohort study on this topic analyzed waiting time after CRT in 1593 patients; the pCR rate was highest in patients waiting 10 to 11 weeks interval.^[28]^ One meta-analysis in 2013 including 13 trials, 3584 patients concluded that an interval longer than 6 to 8 weeks from the end of neoadjuvant CRT and surgery significantly improved pCR rate compared to shorter interval group around 6% (19.5% vs 13.5%).^[29]^ Recently, a prospective randomized trial (GRECCAR-6 Trial) reported that waiting interval more than 11 weeks after CRT did not increase pCR rate after surgery.^[30]^ Due to these conflicting results, there was no specific waiting time period recommended in guidelines. The wide range of 8 to 12 weeks was suggested as an appropriate interval time after CRT.^[31]^ In our study waiting time in pCR and non-pCR group was different (74 days vs 63 days) even without statistically significant. However, in the group of patients who had waiting time >11 weeks, half of them achieved pCR. There is no logically explanation for this result but it could be explored more in future and larger size study.

Tumor length >4 cm is the only strong predictive factor correlated with decreased rate of pCR in our study. There were few previous studies reported that tumor length was the predictor for pCR. Ren et al in 2019 showed that tumor length ≤3 cm and well differentiation tumor were the significant factors associated with pCR.^[18]^ Ouyang et al reported in 2021 that tumor length may be an early predictor of pCR and high sensitivity to total neoadjuvant treatment. However, this study did not give the cut-off point of tumor length. The major limitation in this study is 43% of patients received mFOLFOX6 which is not standard regimen for CRT.^[20]^ The major advantage of tumor length as a predictive factor for pCR is it can be obtained in preoperative period, thus helping surgeons for preoperative decision making before treatment. Regarding to our results, we can establish some recommendations. First, low rectal cancer patients who had preoperative tumor length ≤4 cm may has a chance to be the potential candidate of watch and wait strategy due to high rate of pCR, these might be benefit if they refused to proceed with abdominoperineal resection. Second, locally advanced rectal cancer patients who had preoperative tumor length >4 cm may require additional treatment in order to improve pCR rate and oncological outcome. Longer waiting time between 11 and 12 weeks and more intense neoadjuvant therapy may be considered as the potential options. Futuremore, large scale randomized controlled trial will be the next step to answer this question.

Positive lymph node status was only associated with decreased pCR on univariate analysis, but not on multivariate analyses in our study. Previous studies have shown that positive nodal status is one factor negatively correlated with pCR suggesting that this correlation might be a real effect but not shown up due to small size of our study.^[15,19]^

Interestingly, one patient (8.3%) in our study had residual cancer in perirectal lymph nodes (ypT0N+) even they achieved pCR of primary tumor. This result is higher than the previous reported of 5% incidence of positive lymph nodes in ypT0 patients after CRT.^[32]^ Remaining nodal disease will eventually lead to higher local recurrence, or even distant metastasis. Local excision or watch and wait protocol of rectal cancer patients after achieved cCR is still controversial.^[32,33]^ Because there are no reliable methods to confirm N0 status after CRT, therefore radical surgery is recommended for all patients who had achieved cCR after CRT.

Overall loco-regional and distant failures in our study was 30.6%. Lung metastasis was the most common (34.6%). We have only 2 patients with local recurrence (7.7%) and later found to have distant metastasis. This result of local recurrence rate was comparable to other studies which was ranged between 2.4% and 11%.^[7,34–36]^

We acknowledge several limitations of our study. First the number of patients was relatively small and from single institution so it could limit generalizability of results. Second, few number of patients had endoscopic assessment for clinical response in the earlier year of our study because at that time there was no standard protocol for re-evaluation after CRT in our institution. However, in the last 2 years of the study, there was an implementation of standard protocol after CRT, so the overall rate of endoscopic assessment of clinical response during this period approached 100%. Third, although the most common chemotherapy regimen used was 5FU+LV (81.2%), there were up to three chemotherapy regimens used in our study which may affect clinical and pathological response of the tumors. Fourth, there were 14 patients (16.4%) who had postoperative infectious complications which can result in delayed postoperative chemotherapy. Given on these limitations, our study has several strengths. Eighty from 85 patients (94.1%) were operated by single colorectal surgeon; result in the uniform of surgical techniques. Additionally, there were no major anastomosis leakage encountered except 3.4% rate of presacral collection which were successfully treated with percutaneous drainage so these did not affect the postoperative adjuvant schedule. Furthermore, our mean follow-up time was 65 months which is long enough to reveal the trend of difference in DFS and OS between pCR and non-pCR groups.

## 5 Conclusion

The findings of our study suggested that tumor length >4 cm was the only factor significantly associated with decreased rate of pCR on univariate and multivariate analyses whereas others did not. This finding should be explored in future, large scale prospective studies as there may be implications for selection of appropriate treatment in these group of rectal cancer patients with tumor length longer than 4 cm such as longer waiting time or more intense neoadjuvant treatment to improve pCR rate and long-term oncological outcome.

## Author contributions

**Conceptualization:** Weerapat Suwanthanma, Saowanee Kitudomrat, Chakrapan Euanorasetr.

**Data curation:** Weerapat Suwanthanma, Saowanee Kitudomrat.

**Formal analysis:** Weerapat Suwanthanma, Saowanee Kitudomrat.

**Methodology:** Saowanee Kitudomrat, Chakrapan Euanorasetr.

**Project administration:** Weerapat Suwanthanma, Saowanee Kitudomrat, Chakrapan Euanorasetr.

**Resources:** Weerapat Suwanthanma.

**Supervision:** Weerapat Suwanthanma.

**Writing – original draft:** Weerapat Suwanthanma, Saowanee Kitudomrat, Chakrapan Euanorasetr.

**Writing – review & editing:** Weerapat Suwanthanma, Saowanee Kitudomrat, Chakrapan Euanorasetr.
